# A Systematic Inflammation-based Model in Advanced Pancreatic Ductal Adenocarcinoma

**DOI:** 10.7150/jca.30561

**Published:** 2019-10-22

**Authors:** Li-Xia Wu, Xiao-Yong Wang, Ke-Qun Xu, Yu-Li Lin, Wen-Yu Zhu, Long Han, Yue-Ting Shao, Han-Yu Zhou, Hua Jiang, Jun-Jie Hang, Xu-Guang Yang

**Affiliations:** 1 Department of Oncology, Shanghai Cancer Institute, Renji Hospital, School of Medicine, Shanghai Jiao Tong University, Pujian Road 160, Shanghai 200127, China; 2 Department of Oncology, Shanghai JingAn District ZhaBei Central Hospital, Zhonghuaxin Road 619, Shanghai 200040, China; 3 Department of Gastroenterology, Changzhou No.2 People's Hospital, Affiliated Hospital of Nanjing Medical University, Xinglong Road 29, Changzhou, Jiangsu 213003, China; 4 Department of Oncology, Changzhou No.2 People's Hospital, Affiliated Hospital of Nanjing Medical University, Xinglong Road 29, Changzhou, Jiangsu 213003, China; 5 Department of Immunology, School of Basic Medical Sciences, Fudan University, Shanghai 200030, China; 6 Cancer Institute, Longhua Hospital, Shanghai University of Traditional Chinese Medicine, South Wanping Road 725, Shanghai 200000, China

**Keywords:** pancreatic ductal adenocarcinoma, inflammatory biomarkers, nomogram, prognosis

## Abstract

Emerging evidence revealed the critical role of systematic inflammation in pancreatic ductal adenocarcinoma (PDAC). In the present study, we reviewed the records of 279 patients with advanced PDAC. Among them, 147 cases were used as the training cohort and another 132 as the validation cohort. In the training cohort, distant metastasis, carbohydrate antigen 19-9 (CA19-9), Glasgow prognostic score (GPS), neutrophil-to-lymphocyte ratio (NLR), and lymphocyte-to-monocyte ratio (LMR) were independent prognostic factors in Cox regression. A nomogram based on these factors was generated to predict median survival time and survival probabilities at 6, 12, and 18 months. The nomogram showed a better discriminatory ability than the American Joint Committee on Cancer (AJCC) TNM staging (C-index: 0.727 vs. 0.610). In the validation cohort, a nomogram composed of the same variables also showed a high discriminatory ability (C-index: 0.784). In the low-risk group with a nomogram total point (NTP) value of more than 175, patients receiving combination therapy showed better prognosis than those receiving monotherapy (*P*=0.015). In conclusion, the nomogram based on inflammatory biomarkers can serve as useful prognostic tool for advanced PDAC. In addition, patients with high NTP can greater benefit from combination chemotherapy than monotherapy.

## Introduction

PDAC is a “silent killer” worldwide with an extremely poor prognosis^1^. Most patients with PDAC are asymptomatic and approximately 80% of PDAC cases are diagnosed at a locally advanced or metastatic stage^2^. Although there have been gradual improvements in diagnostic approaches and treatment, such as FOLFIRINOX, the prognosis of PDAC remains dismal^3^. Such condition calls for an urgent need to better discriminate overall survival (OS) at diagnosis to provide valuable information for precise decision-making. Thus, it is of vital importance to identify reliable prognostic models that can be used in clinical practice.

The host immune system is crucial in the pathophysiology of PDAC^4^. Recent reports revealed complex interactions between cancer cells and immune cells, which can regulate tumor growth, progression, metastasis, and angiogenesis^5^. Moreover, inflammatory biomarkers, such as C-reactive protein (CRP), albumin, GPS, NLR, LMR, and platelet-to-lymphocyte ratio (PLR) can serve as prognostic factors for OS in pancreatic cancer^6^-^11^. Most of these biomarkers were evaluated separately and debate continues about the accuracy and validity of predicting prognosis with single parameters.

Staging based on the tumor, node, and metastasis system is the most widely used tool for routine prognostication and treatment of PDAC. However, other factors such as patients' performance status and nutritional status also affect the prognosis of PDAC, which may cause large variations in clinical outcome in patients with the same TNM stage. Such limitations of the AJCC TNM staging system may lead to inaccurate predictions of prognosis and inappropriate treatment strategies in clinical practice.

Nomograms are simple graphical tools integrating diverse variables for determining personalized medicine^12^. They have been commonly used to estimate prognosis in various tumors. Thus, the aim of this study was to investigate the prognostic and predictive value of a nomogram based on inflammatory biomarkers and compare it with the AJCC TNM staging system.

## Patients and Methods

### Patients

Retrospective analyses were conducted in 147 patients enrolled at Shanghai Renji Hospital and 132 patients at Changzhou No.2 People's Hospital with locally advanced or metastatic pancreatic cancer (ICD, Tenth Revision, codes C25). The following inclusion criteria were applied: (1) pathologically confirmed pancreatic ductal adenocarcinoma, either by surgical resection or needle biopsy; (2) locally advanced unresectable or metastasis disease diagnosed by computed tomography (CT) or magnetic resonance imaging (MRI); (3) at least two cycles of palliative chemotherapy after the first diagnosis; and (4) available clinical data at the time of first diagnosis. Patients received first-line chemotherapy regimens including gemcitabine monotherapy, gemcitabine combination therapy (including gemcitabine and S-1 combination therapy, gemcitabine and nab-paclitexal combination therapy) and gemcitabine exclusive therapy (including S-1 monotherapy and FOLFIRINOX)^13^-^15^. In total, 73 patients received monotherapy and 74 patients received combination therapy in the training cohort. Meanwhile, 75 patients received monotherapy and 57 patients received combination therapy in the validation cohort. Ethical approval was obtained *by* the ethics committees of Changzhou No.2 People's Hospital. The methods were carried out in accordance with the principles of Declaration of Helsinki.

### Prognostic factors

Fifteen clinical variables including patients' demographics, medical treatment records, pathological reports, and pretreatment laboratory data were collected for analysis (Table S1). The GPS was determined as follows: patients with high CRP levels (>10 mg/L) and low albumin levels (<35 g/L) were scored 2, those with either abnormality were given a score of 1, and those without any abnormal values were given a score of 0^16^.

### Statistical analysis

Statistical analyses were conducted with R 3.3.1 software (Institute for Statistics and Mathematics, Vienna, Austria) and SPSS statistical software (version 21.0, SPSS Inc, IBM, Armonk, NY, USA). The optimal cutoff values of NLR, LMR and PLR were identified by generating receiver operating characteristics (ROC) curves^17^. Chi-square test for trend was used to evaluate the relationship between GPS and clinicopathological characteristics. The correlations between NLR, LMR, PLR, and clinicopathological characteristics were assessed by Chi-square test and Continuity Correction. OS was defined as the date from chemotherapy initiation to the date of death for any reason or censored on the last follow-up visit. In the training cohort, independent prognostic factors for OS were investigated using the Cox regression model. These prognostic factors were further used to generate the nomogram. The discrimination of the nomogram was evaluated by the C-index^18^. In addition, the calibration plot was used to assess the probability of concordance between predicted survival with actual survival. The stratification of OS via NTP was demonstrated by Kaplan-Meier analysis. We confirmed the superiority of the nomogram over AJCC TNM staging system by calculating the C-index of both. We demonstrated heterogeneity within the AJCC staging system predictions by generating a histogram of nomogram-predicted probability. The discriminatory ability of the nomogram was also externally validated by the validation cohort. Two-sided *P*<0.05 was considered statistically significant in all tests.

## Results

### Patients' characteristics

Table S1 details the baseline clinicopathological characteristics of patients with advanced PDAC in both the training and validation cohorts. Patients were divided into groups according to GPS (0, 1, and 2), NLR (≥2.8 or <2.8), LMR (≥2.8 or <2.8), and PLR (≥192.2 or <192.2). Then baseline clinicopathological characteristics were compared between these groups in the training cohort (Table 1). GPS was found to be significantly correlated with distant metastasis (*P*=0.005). A statistically significant association of NLR was observed with gender (*P*=0.009) and distant metastasis (*P*<0.001). In addition, gender (*P*=0.001), ECOG PS (*P*=0.015), distant metastasis (*P*<0.001) and CA19-9 (*P*=0.023) were associated with LMR.

### Comparison of OS stratified by GPS, NLR, LMR and PLR

In the training cohort, Kaplan-Meier analysis showed that the median OS was 10.6 months in the GPS = 0 group, 6.5 months in the GPS = 1 group, and 2.8 months in the GPS = 2 group (*P*<0.001; Figure S1A). Likewise, median OS in patients with a pretreatment NLR<2.8 was 11.0 months, which was significantly longer than that of patients with an NLR≥2.8 (5.3 months) (P<0.001; Figure S1B). In addition, patients with LMR<2.8 had poorer OS compared with those with LMR≥2.8 (5.2 vs. 10.8 months) (*P*<0.001; Figure S1C). However, median OS was comparable between the two groups identified by PLR (8.4 vs. 8.9 months, *P*=0.333; Figure S1D).

### Prognostic factors for OS

**Figure 1:**
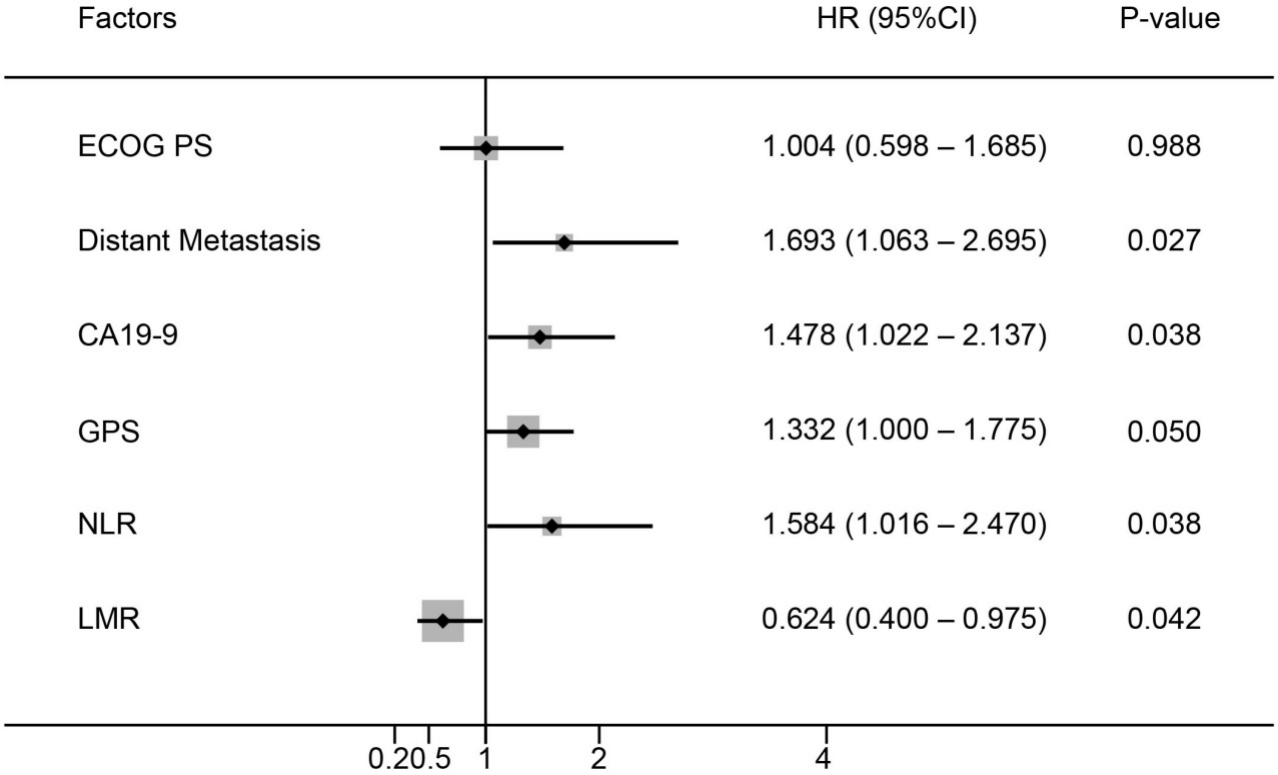
Multivariate analysis of prognostic factors for OS in patients with advanced PDAC.

Six baseline characteristics were correlated with OS in univariate analysis in the training cohort (Table 2). These included Eastern Cooperative Oncology Group performance status (ECOG PS), distant metastasis, CA19-9, GPS, LMR and NLR. Among them, five factors including distant metastasis (*P*=0.027), CA19-9 (*P*=0.038), GPS (*P*=0.050), LMR (*P*=0.038) and NLR (*P*=0.042) showed independent prognostic value in the multivariate analysis (Figure 1).

### Establishment and validation of the nomogram

**Figure 2:**
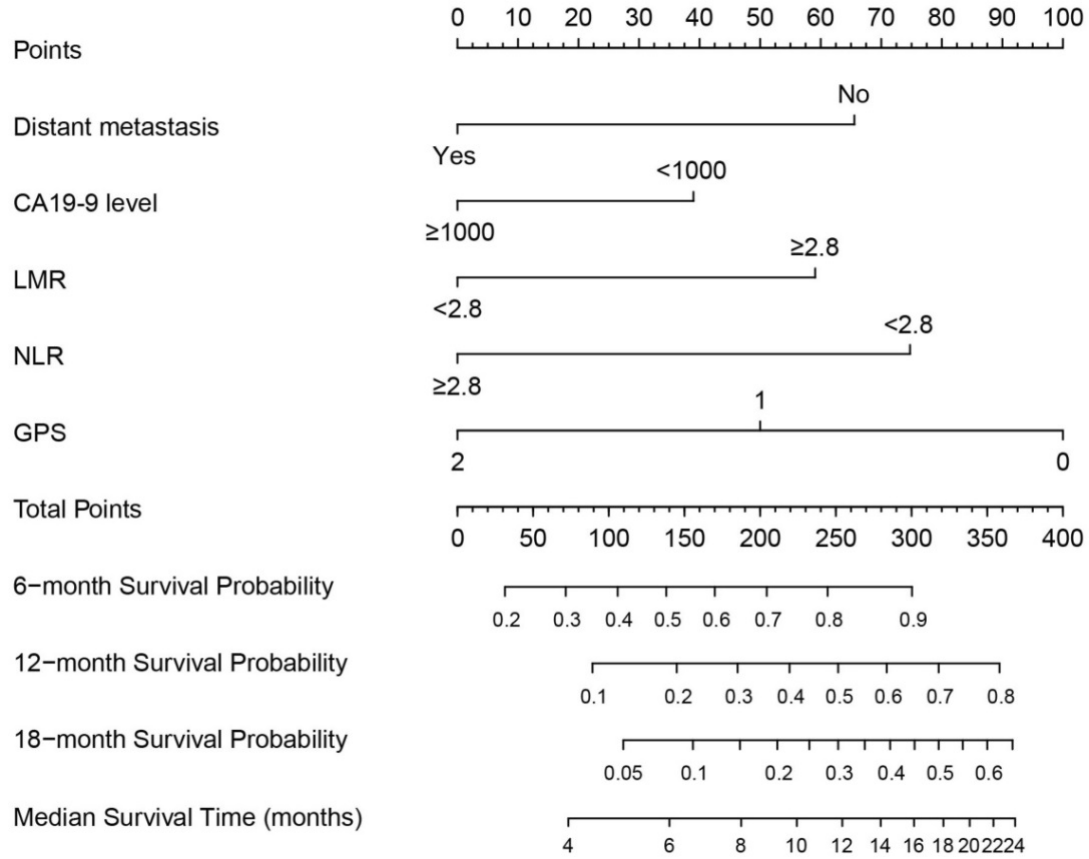
Prognostic nomogram for predicting 6-, 12- and 18-month OS probability based on distant metastasis, CA19-9 level, LMR, NLR and GPS in patients with advanced PDAC.

**Figure 3:**
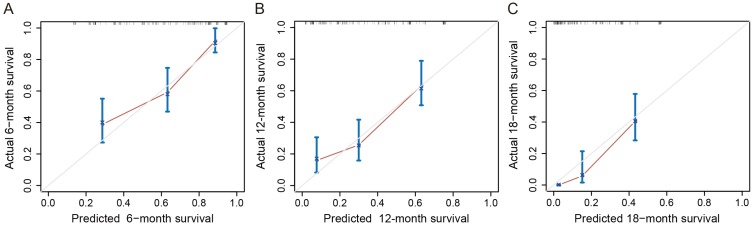
Calibration curves of the nomogram for predicting survival probabilities at 6 (A), 12 (B), and 18 (C) months. The diagonal line: the ideal calibrated model. Black line: actual calibration. Circles: median. X: mean. 95% CIs are depicted for each point along the calibration curve.

A nomogram was constructed based on these prognostic factors in the training cohort (Figure 2). This nomogram could predict patients' median survival time and survival probabilities at 6, 12 and 18 months. The Harrell's C-index of the nomogram was 0.727. After adjustment by bootstrapping with 1,000 re-samples, the calibration plots, which showed concordance between the actual and the ideal survival predictions, were demonstrated for 6-month, 12-month and 18-month survival (Figure 3). Meanwhile, a nomogram composed of the same variables in the external validation cohort also showed a good discriminatory ability (C-index: 0.784).

### Prognostic score for OS stratification and therapeutic decision-making

**Figure 4:**
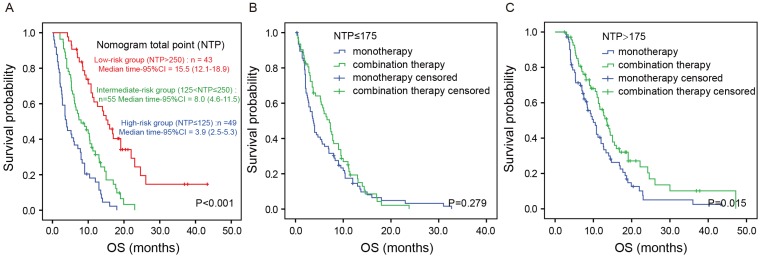
Kaplan-Meier analysis according to the NTP-based groupings in the training cohort. Kaplan-Meier analysis according to the NTP-based groupings (A). Kaplan-Meier analysis based on the chemotherapy regimens in the high-risk group (B) and low-risk group (C).

NTP was calculated by summing the “point” value of the five prognostic factors. Based on NTP, the patients were categorized into three groups: a low-risk group (NTP>250), an intermediate-risk group (125<NTP≤250) and a high-risk group (NTP≤125). Figure 4A shows the Kaplan-Meier analysis according to the NTP-based groupings. OS was distinctly different between the three groups (*P*<0.001). The median OS was 3.9 (95%CI: 2.5-5.3) months in the high-risk group, 8.0 (95% CI：4.6-11.5) months in the intermediate-risk group and 15.5 (95%CI: 12.1-18.9) months in the low-risk group (*P*<0.001). To investigate the utility of this nomogram in therapeutic decision-making, we divided all the patients into two groups according to NTP: a low-risk group (NTP>175) and a high-risk group (NTP≤175). Intriguingly, in the high-risk group, there was no significant difference in OS between patients receiving monotherapy or combination therapy (*P*=0.279; Figure 4B). In contrast, in the low-risk group, patients receiving combination therapy showed better prognosis than those receiving monotherapy (median OS 13.5 vs. 10.0 months, *P*=0.015; Figure 4C). In accordance with the finding in the training cohort, the result was similar in the validation cohort (Figure S2).

### AJCC TNM stage and nomogram-predicted survival probabilities

**Figure 5:**
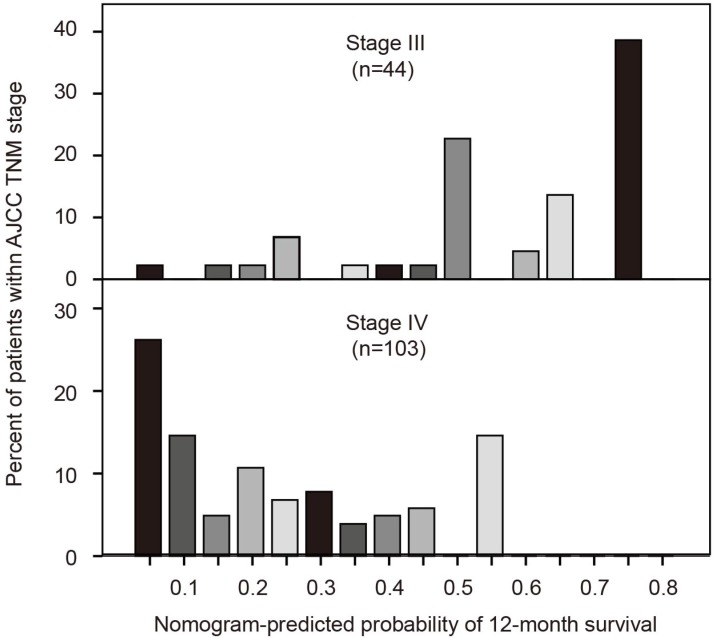
Comparisons of nomogram predictions with that of AJCC TNM staging groupings.

The seventh edition AJCC TNM system uses tumor, lymph node and metastasis for grouping but is without precise discriminatory ability for advanced pancreatic cancer. We developed a histogram of nomogram-predicted probability of 12-month survival for stages III and IV. Notably, even for the same TNM stage, there was considerable heterogeneity in the nomogram-predicted probabilities (Figure 5). In addition, the nomogram composed of both clinicopathological characteristics and inflammatory biomarkers showed a better discriminatory ability than AJCC TNM system (C-index: 0.727 vs. 0.610).

## Discussion

PDAC is a lethal disease and its prognosis is affected by a variety of factors including the host immune system. Currently, the TNM staging system remains the gold standard in oncology for both diagnosis and prognostication^19^. However, the system has several limitations and is unable to integrate tumor, nodes, and metastases as continuous variables without incorporating variables that affect prognosis such as inflammatory biomarkers^12^. For advanced PDAC, the TNM staging system only depends on distant metastasis to dichotomise patients into stage III or IV, resulting in poor discrimination and making it difficult for clinicians to determine a precise treatment course. Thus, we investigated whether a nomogram based on inflammatory biomarkers could predict survival for PDAC more accurately.

Nomograms are user-friendly graphical tools that incorporate diverse prognostic variables into prognosis. Currently, nomograms are progressively being used in estimating prognosis in oncology and in the move towards personalized medicine. Using nomograms, cancer patients can be evaluated and stratified in clinical trials to ensure well-balanced arms. Several nomograms have been developed for pancreatic cancer. In 2004, Brennan et al. first constructed a nomogram to predict survival probabilities in patients who underwent resection for PDAC and made it available online^20^. An external validation by Ferrone et al. in 2005 further proved the prognostic value of nomogram^21^. Because these two studies mainly focused on patients with resectable PDAC, Hamada et al. found that nomograms could provide valuable information for tailored decision-making early after the diagnosis of nonresectable pancreatic cancer^22^. In addition, on the basis of the largest phase III clinical trial of locally advanced pancreatic cancer, Vernerey et al. developed and validated a prognostic nomogram and a score for OS in locally advanced pancreatic cancer^23^. However, none of these nomograms were composed of classical inflammatory biomarkers while the fact that systematic inflammation can affect cancer patients' prognosis has long been recognized.

Recently, inflammatory biomarkers have demonstrated their prognostic value in patients with PDAC. These biomarkers included CRP, albumin, lymphocyte, monocyte, neutrophil, platelet and their derivatives- GPS, LMR, NLR, and PLR 24-27. However, most previous studies mainly focused on one of these biomarkers and the results were controversial. In the present study, apart from distant metastasis and CA19-9, we found GPS, NLR and LMR to be independent prognostic factors in advanced PDAC but PLR showed no prognostic value. To predict survival for advanced PDAC more accurately, we added these three inflammatory biomarkers to the nomogram and found it showed a better discriminatory ability than one without inflammatory biomarkers (C-index: 0.727 vs. 0.638). The mechanism of the prognostic values of these inflammatory biomarkers in advanced PDAC remains to be illustrated. For example, circulating monocytes are the major origin of tumor-associated macrophages, which orchestrate various aspects of cancer including the diversion and skewing of adaptive responses, angiogenesis, cell proliferation, matrix remodeling and the construction of a metastatic niche^28^.

In clinical practice, only a few indexes, such as ECOG PS, can be used to determine whether patients with advanced PDAC should receive monotherapy or combination therapy. However, these indexes only revealed one aspect of patients while the final effect of chemotherapy can be affected by various factors such as nutritional status, immune function of patients and the biological characteristics of cancer. In our current study, we found that, in the low-risk group (NTP>175) rather than the high-risk group (NTP≤175), patients receiving combination therapy showed better prognosis than those receiving monotherapy (median OS 13.5 vs. 10.0 months, *P*=0.015). Because the nomogram included distant metastasis, CA 19-9 level, LMR, NLR and GPS, which could comprehensively reveal nutritional status and immune function of patients, as well as the biological characteristics of cancer, such a result suggested that patients with better nutritional status, immune function, and a less invasive tumor may benefit more from combination therapy.

As previous studies reported, we also found that the nomogram composed of both clinicopathological characteristics and inflammatory biomarkers showed a better discriminatory ability than the AJCC TNM system. The Harrell's C-index of AJCC TNM staging was 0.610 in our research, which was similar to that of other reports (Brennan et al., 0.56; Ferrone et al., 0.59; and Hamada et al., 0.612)^20^-^22^. However, the present nomogram demonstrated a better discriminatory ability (Harrell's C-index, 0.727), which was slightly higher than previously reported nomograms in pancreatic cancer (Brennan et al., 0.64; Ferrone et al., 0.62; Hamada et al., 0.686; and Vernerey et al., 0.68)^20^-^23^. This indicated that the addition of inflammatory biomarkers to the nomogram may increase its discriminatory performance. Figure 5 also demonstrates the considerable heterogeneity within AJCC TNM stages III and IV. Thus, for pancreatic cancer patients with the same TNM stage, physicians can more precisely predict prognosis and adopt more individualized management.

Several limitations should be addressed in this study. First, the study was limited by its retrospective design and a rather small population size. Second, there was a large degree of heterogeneity in the chemotherapy regimens received by patients, which may affect the robustness of the results. In addition, other factors that may influence survival such as tumor size were not included in this model. Therefore, further prospective studies with large sample sizes and external validation are needed to validate the prognostic value of nomograms composed of inflammatory biomarkers in PDAC.

In conclusion, a nomogram based on inflammatory biomarkers can serve as useful prognostic tool for advanced PDAC. In addition, patients with high NTP can benefit more from combination chemotherapy than monochemotherapy.

## Supplementary Material

Supplementary figures and tables.

## Figures and Tables

**Table 1:** Comparison of baseline characteristics according to GPS, NLR, LMR and PLR

Characteristics	GPS (n)			P	NLR (n)		P	LMR (n)		P	PLR (n)		P
	0	1	2		<2.8	≥2.8		<2.8	≥2.8		<192.2	≥192.2	
**Gender**													
Male	45	38	14	0.191	42	55	0.009	54	43	0.001	75	22	0.709
Female	29	16	5		33	17		14	36		40	10	
**Age**													
<60	34	28	3	0.115	30	35	0.293	32	33	0.520	51	14	0.952
≥60	40	26	16		45	37		36	46		64	18	
**ECOG PS**													
2	8	12	3	0.245	9	14	0.214	16	7	0.015	16	7	0.273
0-1	66	42	16		66	58		52	72		99	25	
**Primary tumor location**													
Head and neck	30	23	8	0.845	34	27	0.335	24	37	0.157	50	11	0.355
Body and tail	44	31	11		41	45		44	42		65	21	
**Distant metastasis**													
Yes	45	42	17	0.005	42	61	<0.001	60	43	<0.001	81	23	0.874
No	29	12	2		33	11		8	36		34	9	
**CA19-9 (U/ml)**													
<1000	48	28	10	0.166	48	38	0.167	33	53	0.023	67	19	0.910
≥1000	26	26	9		27	34		35	26		48	13	
**CEA (ng/ml)**													
<5	31	16	8	0.558	32	23	0.179	25	30	0.880	44	11	0.688
≥5	43	38	11		43	49		43	49		71	21	
**Hemoglobin (g/L)**													
<100	3	4	3	0.102	3	7	0.168	7	3	0.119	7	3	0.798
≥100	71	50	16		72	65		61	76		108	29	

**Table 2:** Univariate analysis of factors for OS in patients with advanced PDAC

Characteristics	HR	95%CI	P
**Gender**			
Male	0.891	0.610-1.302	0.552
Female			
**Age**			
<60	0.921	0.649-1.307	0.644
≥60			
**ECOG PS**			
2	1.909	1.206-3.023	0.006
0-1			
**Primary tumor location**			
Head and neck	1.171	0.821-1.670	0.384
Body and tail			
**Distant metastasis**			
Yes	2.701	1.780-4.098	<0.001
No			
**CA19-9 (U/ml)**			
≥1000	1.753	1.219-2.521	0.002
<1000			
**CEA (ng/ml)**			
≥5	1.285	0.890-1.853	0.180
<5			
**Hemoglobin (g/L)**			
<100	0.829	0.403-1.702	0.609
≥100			
**GPS**	1.838	1.437-2.352	<0.001
0			
1			
2			
**NLR**			
NLR≥2.8	2.860	1.985-4.121	<0.001
NLR<2.8			
**LMR**			
LMR≥2.8	0.342	0.237-0.495	<0.001
LMR<2.8			
**PLR**			
PLR<192.2	0.804	0.515-1.256	0.338
PLR≥192.2			

## Abbreviations

PDAC: pancreatic ductal adenocarcinoma
CA19-9: carbohydrate antigen 19-9
GPS: Glasgow prognostic score
NLR: neutrophil-to-lymphocyte ratio
LMR: lymphocyte-to-monocyte ratio
AJCC: American Joint Committee on Cancer
NTP: nomogram total point
OS: overall survival
CRP: C-reactive protein
PLR: platelet-to-lymphocyte ratio
CT: computed tomography
MRI: magnetic resonance imaging
ROC: receiver operating characteristics
ECOG PS: Eastern Cooperative Oncology Group performance status
