# Supplementary material for: A Systematic Inflammation-based Model in Advanced Pancreatic Ductal Adenocarcinoma
Source: J Cancer. 2019 Oct 22;10(26):6673–80. doi: 10.7150/jca.30561 (PMC6856880; doi:10.7150/jca.30561)
Supplement: Supplementary file 1 — Supplementary figures and tables. [file jcav10p6673s1.pdf]

Supplement materials

Table S1. Baseline characteristics of patients with advanced PDAC

Valuables	Category	Training cohort	Validation cohort
Gender	Male	97 (66.0%)	74 (56.1%)
	Female	50 (34.0%)	58 (43.9%)
Age	Median (Range)	60 (34-84)	64 (36-85)
ECOG PS	0	14 (9.5%)	16 (12.1%)
	1	110 (74.8%)	47 (35.6%)
	2	23 (15.6%)	69 (52.3%)
Primary tumor location	Head and neck	61 (41.5%)	63 (47.7%)
	Body and tail	86 (58.5%)	69 (52.3%)
Distant metastasis	No	43 (29.3%)	17 (12.9%)
	Yes	104 (70.7%)	115 (87.1%)
CRP	Median (Range)	4.0 (0.2-178.0)	3.5 (0.1-185.0)
Albumin	Median (Range)	38.7 (26.1-48.4)	37.0 (19.5-53.4)
ALC (*10^9/L)	Median (Range)	1.30 (0.29-4.87)	1.37 (0.43-3.74)
AMC (*10^9/L)	Median (Range)	0.46 (0.10-1.62)	0.40 (0.01-3.50)
ANC (*10^9/L)	Median (Range)	3.74 (1.01-14.07)	4.10 (0.96-26.3)
PLT (*10^9/L)	Median (Range)	178.0 (37.0-641.0)	185.0 (17.0-763.0)
CA19-9 (U/ml)	Median (Range)	474.0 (0.6-2084.0)	900.5 (0.1-2068.0)
CEA (ng/ml)	Median (Range)	7.4 (0.4-1065.0)	5.2 (0.6-1037.0)
Hemoglobin (g/L)	Median (Range)	121 (75-168)	120 (73-153)

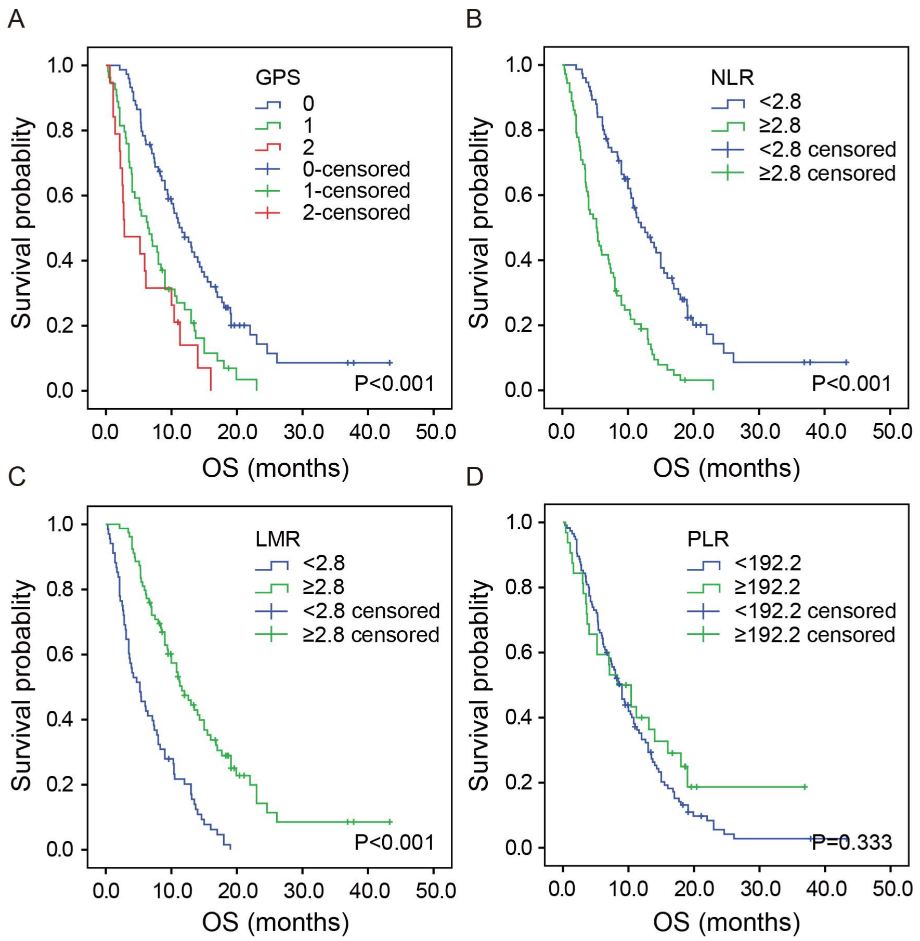

Figure S1. Kaplan-Meier estimates of overall survival according to GPS (A), NLR (B) LMR (C) and PLR (D).

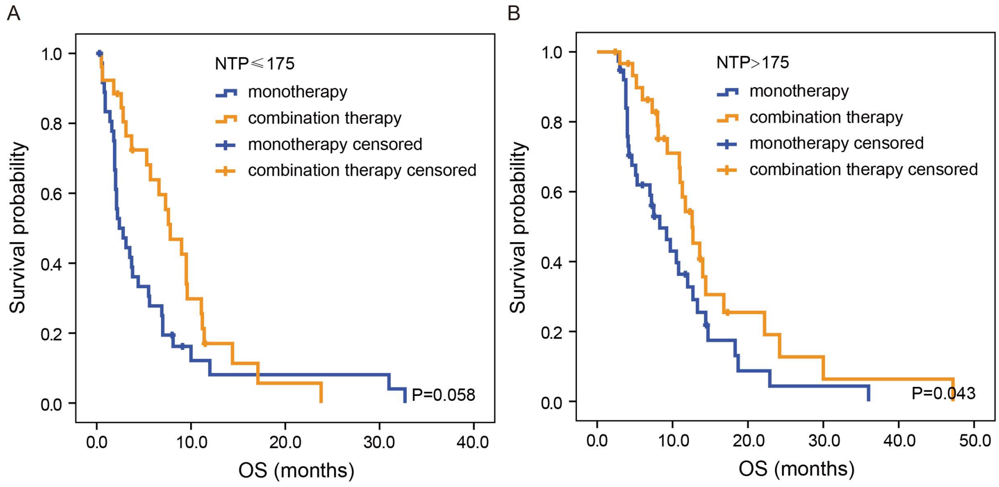

Figure S2. Kaplan-Meier analysis according to the chemotherapy regimens in the validation cohort. Kaplan-Meier analysis based on the chemotherapy regimens in the high-risk group (A) and low-risk group (B).
